# Effects of Al interlayer coating and thermal treatment on electron emission characteristics of carbon nanotubes deposited by electrophoretic method

**DOI:** 10.1186/1556-276X-9-236

**Published:** 2014-05-13

**Authors:** Bu-Jong Kim, Jong-Pil Kim, Jin-Seok Park

**Affiliations:** 1 Department of Electronic Systems Engineering, Hanyang University, Ansan, Gyeonggi-do 426-791, Republic of Korea; 2 R&D Team, Health & Medical Equipment Business Team, Samsung Electronics, Suwon, Gyeonggi-do 443-742, Republic of Korea

**Keywords:** Carbon nanotube (CNT), Electrophoretic deposition (EPD), Al interlayer coating, Thermal treatment, Field electron emission, Emission stability

## Abstract

The effects of aluminum (Al) interlayer coating and thermal post-treatment on the electron emission characteristics of carbon nanotubes (CNTs) were investigated. These CNTs were deposited on conical-shaped tungsten (W) substrates using an electrophoretic method. The Al interlayers were coated on the W substrates via magnetron sputtering prior to the deposition of CNTs. Compared with the as-deposited CNTs, the thermally treated CNTs revealed significantly improved electron emission characteristics, such as the decrease of turn-on electric fields and the increase of emission currents. The observations of Raman spectra confirmed that the improved emission characteristics of the thermally treated CNTs were ascribed to their enhanced crystal qualities. The coating of Al interlayers played a role in enhancing the long-term emission stabilities of the CNTs. The thermally treated CNTs with Al interlayers sustained stable emission currents without any significant degradation even after continuous operation of 20 h. The X-ray photoelectron spectroscopy (XPS) study suggested that the cohesive forces between the CNTs and the underlying substrates were strengthened by the coating of Al interlayers.

## Background

Recently, field emitters using carbon nanotubes (CNTs) have been utilized as cold electron sources for high-resolution X-ray apparatuses [1-3]. To use CNTs as electron sources, the turn-on electric field that triggers the field-driven electron emission must be low, and the generated emission current level must be high. Simultaneously, the stability of the emission current must be ensured during a long-term operation. Here, CNTs can be prepared on various types of substrates such as flat types and tip types either by direct [4-6] or indirect [7-10] methods. Practically, the indirect methods have certain advantages over the direct methods due to their simpler deposition systems, lower costs, lower processing temperatures, and easier scale-up. However, the indirect methods demonstrate weak adhesion often with the widely utilized metallic substrates [11,12]. Under a prolonged emission condition, CNTs may be removed on substrates due to their weak adhesion. This makes it difficult to obtain uniform and consistent emission currents from the CNT emitter. For this reason, most of the indirect methods have employed flat-type substrates in preparing CNTs. The use of flat-type substrates, on the other hand, would be less desirable than the use of tip-type substrates for the application of CNTs as electron sources for micro-focus X-ray systems [13]. Therefore, the combination of tip-type substrates and indirect deposition methods is recommended for such application of CNTs only if good adhesion and high levels of emitted currents are guaranteed. Regarding this issue, we have suggested the use of interlayer with hafnium (Hf) thin films between CNTs and tungsten (W) tips [14].

This study aims at fabricating tip-type CNT emitters that have good adhesion and illustrate high levels of emission currents. This has been achieved by depositing CNTs on conical-shaped tip-type W substrates via electrophoretic deposition, by coating interlayers with aluminum (Al) thin films and by performing thermal treatment. The effects of thermal treatment as well as Al interlayer coating on the electron emission behavior and long-term emission stability of CNTs have been investigated extensively.

## Methods

The conical-shaped W substrates were prepared by electrochemical etching [15] of 300-μm-diameter W rods so that they would have very sharp apexes of approximately 500 nm in diameter. Prior to the deposition of CNTs, some of the W substrates were coated by 100-nm-thick Al films via RF magnetron sputtering. The CNTs were deposited on the W substrates with or without Al interlayers by using an electrophoretic deposition (EPD) method [7]. As the initial step for the EPD process, carbon nanopowders with a portion of thin multi-walled CNTs (t-MWCNTs) were purified with a magnetic stirrer in a solution containing a 1:1 volume ratio of concentrated nitric and sulfuric acids. The powder was placed in a dispersion medium and in a vessel containing 50 ml of isopropyl alcohol (IPA). The charger material of Mg(NO_3_)_2_ · 6H_2_O (15 mg) was added to this suspension. The CNT solution was then uniformly mixed via sonication for 10 min. The W-tip substrate coated with or without the Al interlayer was used as the cathode electrode, and the titanium nitride (TiN)-coated p-type silicon (Si) wafer was used as the anode electrode. The distance between the two electrodes in the suspension was sustained at 10 mm. The deposition of CNTs was carried out by applying a constant voltage of 80 V (DC) with the deposition time fixed at 40 s. Finally, several of the CNT samples were thermally treated at 600°C for 30 min in an argon (Ar) atmosphere. The identification of the CNT samples considered in this study is listed in Table 1, according to Al interlayer coating and thermal treatment.

**Table 1:** Identification of the CNT emitters considered in this study

**Samples**	**Al interlayer**	**Thermal treatment**	***I***_**max **_**(μA)**	***V***_**on **_**(V)**	***β*** **(×10**^**4**^**)**	***I***_**D**_**/*I***_**G **_**(Raman)**	***I***_**F**_**/*I***_**I**_
CNT-A	Without	No	71	970	4.46	0.59	0.05
CNT-B	Without	Yes	223	770	4.30	0.40	0.29
CNT-C	With	No	89	950	4.54	0.57	0.79
CNT-D	With	Yes	309	820	4.98	0.43	0.97

The electron emission characteristics of the CNT emitters are also summarized.

## Results and discussion

**Figure 1:**
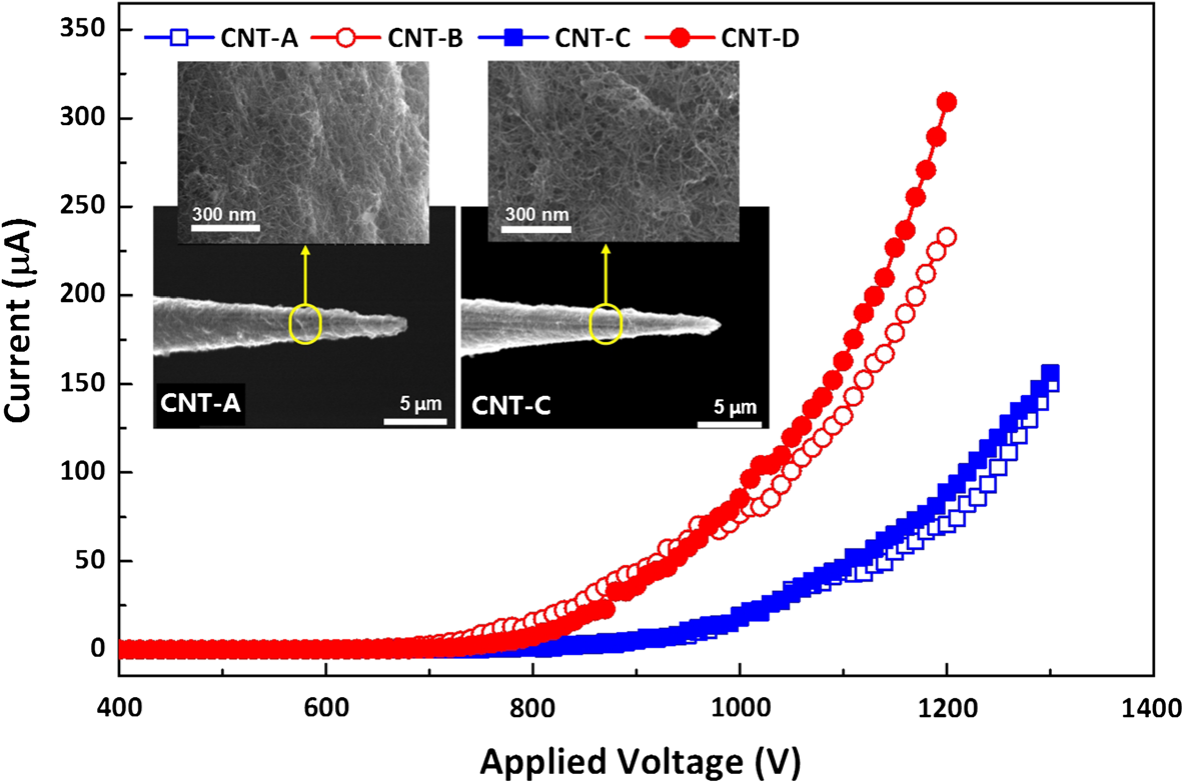
**The emission current versus electric field characteristics of CNTs.** The inserted photos represent the FESEM images of the exterior shapes and CNTs' surfaces for the samples CNT-A and CNT-C.

Figure 1 shows the emission currents of the CNTs, which are listed in Table 1, as a function of the applied voltage. The electron emission characteristics of the deposited CNTs were measured using a compactly designed field emission measurement system. The distance between the cathode (CNT) and the anode (ITO-coated glass) was carefully adjusted to be kept at 1 mm by using a micro-spacing control system. It is clearly seen in Figure 1 that the thermally treated CNTs (i.e., CNT-B and CNT-D) revealed much better emission characteristics than those of the as-deposited CNTs (i.e., CNT-A and CNT-C), while the coating of Al interlayer seems to hardly affect the emission characteristics. From the emission characteristics, the maximum emission current (*I*_max_, μm) and turn-on voltage (*V*_on_, V) of the CNTs were estimated by defining the *I*_max_ as the emission current measured at the applied voltage of 1.2 kV and the *I*_on_ as the voltage applied to obtain the emission current of 10 μA. Also, the field enhancement factor (*β*) values of the CNTs were calculated by applying the emission current characteristics of Figure 1 to the Fowler-Nordheim theory with the work function of CNTs to be 5.0 eV [16]. The values of *I*_max_, *V*_on_, and *β* estimated from all of the CNTs are summarized in Table 1. The results showed that the drastic increase of *I*_max_ and the decrease of *V*_on_ were induced by the thermal treatment of CNTs, regardless of any Al interlayer coating. The *β* values, on the other hand, were not much different from CNT-A to CNT-D and estimated to be within the range from 4.30 × 10^4^ to 4.98 × 10^4^.

For all of the CNTs, the changes in the surface morphologies due to thermal treatment and Al interlayer coating were monitored by using a field emission scanning electron microscope (FESEM; JSM-6330 F, JEOL, Tokyo, Japan). The FESEM images of the exterior shapes and the enlarged surfaces for the CNT-A (without Al interlayer) and CNT-C (with Al interlayer) emitters are compared in Figure 1. It seemed that no significant differences in their surface morphologies were observed. It was also observed in this study that thermal treatment hardly affected the surface morphologies of the CNTs, although their FESEM images are not displayed in Figure 1. This may indicate that neither the coating of Al interlayer nor the thermal treatment altered the structural aspect ratios of the CNTs. Also, this may be in good agreement with the results that the *β* values were similar for all of the CNTs.

**Figure 2:**
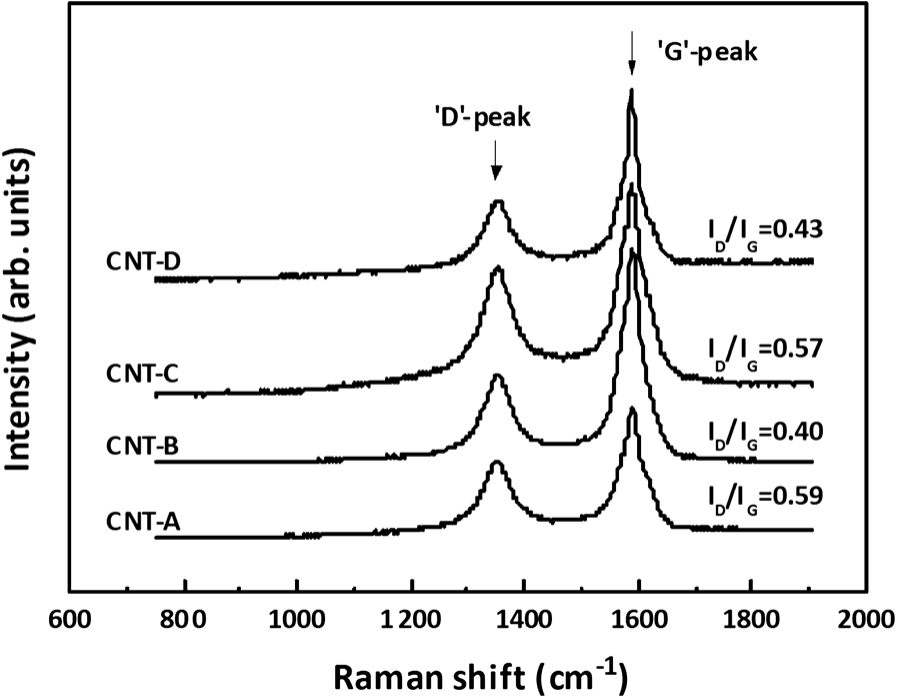
**The Raman spectra of the CNTs.** The estimated *I*_D_/*I*_G_ values are also displayed for all of the CNTs.

To discover any other reason that can account for the results shown in Figure 1, the microstructures of the CNTs were analyzed via Raman spectroscopy (T64000, Jobin Yvon, Edison, NJ, USA). As shown in Figure 2, two typical Raman peaks, the so-called ‘D’ peak (*I*_D_) at around 1,350 cm^-1^ and ‘G’ peak (*I*_G_) at around 1,588 cm^-1^, were observed. The intensity ratios of the two peaks (i.e., *I*_D_/*I*_G_), which has frequently been used to appraise the crystallinity of CNTs [17], were estimated. The resultant *I*_D_/*I*_G_ values, as listed in Table 1, indicated that the *I*_D_/*I*_G_ values were seldom changed by coating of the Al interlayers, but they were significantly reduced by thermal treatment, such as 0.57 to 0.59 for the as-deposited CNTs and 0.40 to 0.43 for the thermally treated CNTs. This may have been because the amorphous carbonaceous by-products, residual binders, and other impurities that were adsorbed on the CNTs' outer walls were somewhat removed during the thermal treatment. Accordingly, it can be inferred from the FESEM and Raman results that the enhanced electron emission of the thermally treated CNTs may be due to the improvement of their crystal qualities [18].

**Figure 3:**
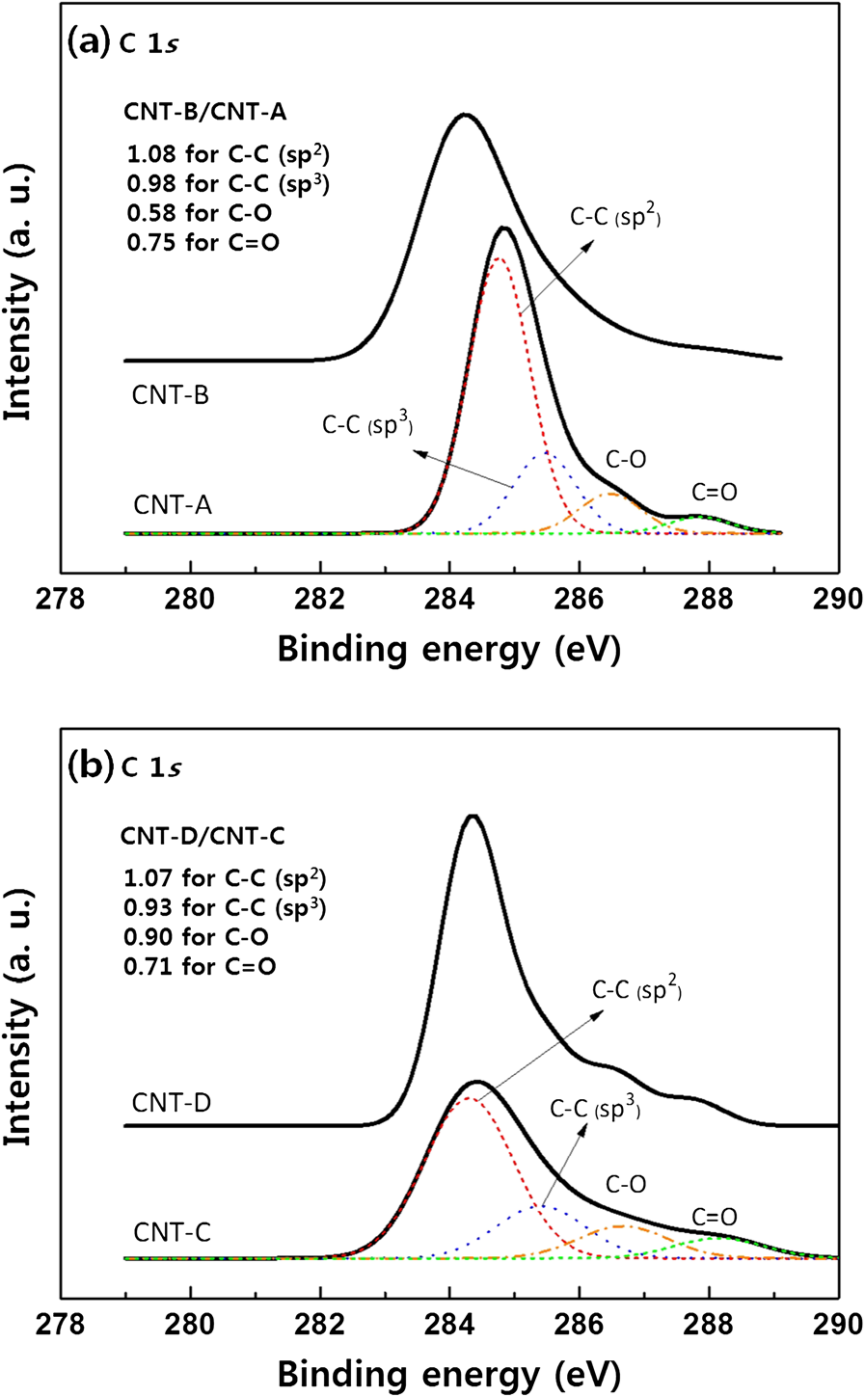
**The XPS spectra for C 1** ***s *states of the CNTs. (a)** The XPS spectra of the CNT-A and CNT-B samples. **(b)** The XPS spectra of the CNT-C and CNT-D samples.

The X-ray photoelectron spectroscope (XPS; MultiLab 2000, Thermo, Pittsburgh, PA, USA) was used to analyze the chemical bonds of the CNTs. Figure 3a,b shows the XPS spectra of the C 1 *s* state for all of the CNT samples. The C 1 *s* spectra were composed of several characteristic peaks, such as two peaks due to the carbon-carbon interactions including C-C *sp*^2^ bonds at the binding energy of 284.4 to 284.7 eV and C-C *sp*^3^ bonds at 285.1 to 285.5 eV, and two relatively weak peaks due to the carbon-oxygen interactions including C-O bonds at 286.4 to 286.7 eV and C = O bonds at 287.8 to 288.1 eV [19]. Also, the variations of the peak intensities due to thermal treatment were calculated, which are expressed in Figure 3a,b as the intensity ratios of thermally treated CNTs (i.e., CNT-B or CNT-D) to as-deposited CNTs (i.e., CNT-A or CNT-C) for each peak (e.g., CNT-B/CNT-A = 1.08 for the C-C *sp*^2^ peak as shown in Figure 3a). The results show that after the thermal treatment, the C-C *sp*^2^ bonds increased, but the C-C *sp*^3^ bonds decreased. This implies the improvement of the CNTs' crystal qualities, which corresponds to the Raman analysis as shown in Figure 2. After the thermal treatment, furthermore, both of the C-O and C = O peaks were observed to be reduced. These carbon-oxygen peaks indicate that oxygen contaminants such as the carbonyl (C = O), carboxyl (-COOH), and hydroxyl (O-H) groups, which may be generated inevitably by acid treatment during the purification process [20], exist in the CNTs. Accordingly, the decrease of the carbon-oxygen peaks in the XPS spectra indicated that the decomposition of the oxygen contaminants occurred via the thermal treatment [21].

**Figure 4:**
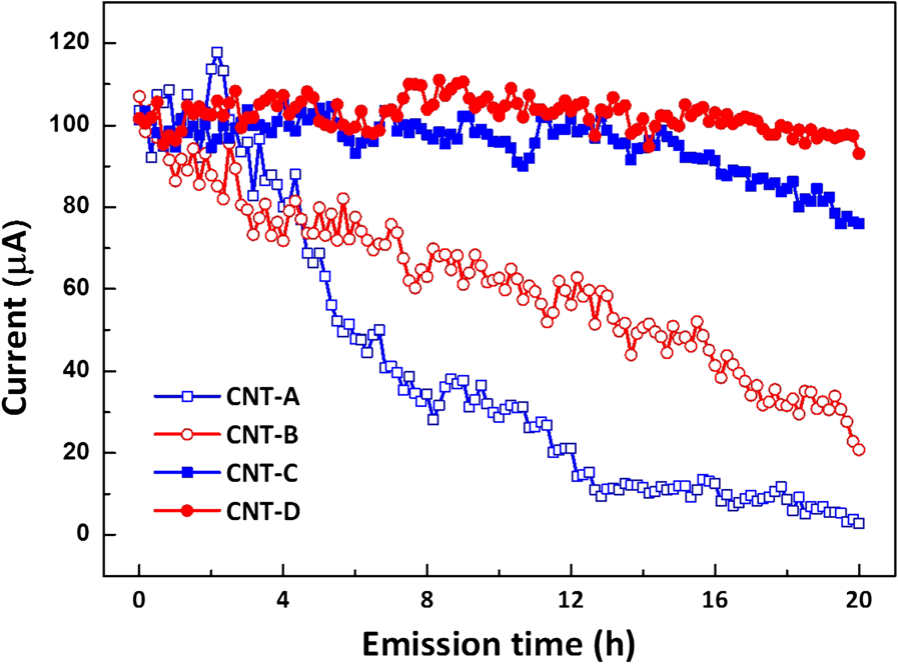
The long-term (20 h) emission characteristics of CNTs.

The following results represent the effects of Al interlayer coating and thermal treatment on the CNTs' electron emission stabilities due to prolonged operation. The stability test was conducted by continuously applying the voltage, which was required for the initial emission current to approach approximately 100 μA, for up to 20 h. The instantaneous emission currents were recorded at 10-min intervals, and the results of the emission stability test are shown in Figure 4. To describe quantitatively the change of emission currents due to the prolonged application of voltage, the average values of the emission currents generated during the initial (0 to 1 h) and final (19 to 20 h) stages of operation (denoted by ‘*I*_I_’ and ‘*I*_F_’, respectively) were calculated, and the ratios of *I*_F_/*I*_I_ are listed in Table 1. As the emission time elapsed, the emission current of the CNTs without Al interlayers (i.e., CNT-A and CNT-B) decreased. At the final stage, the emission currents decreased down to approximately 5% for CNT-A and 29% for CNT-B, as compared with the initial emission currents. On the other hand, the CNTs with Al interlayers (i.e., CNT-C and CNT-D) showed highly stable electron emission characteristics.

**Figure 5:**
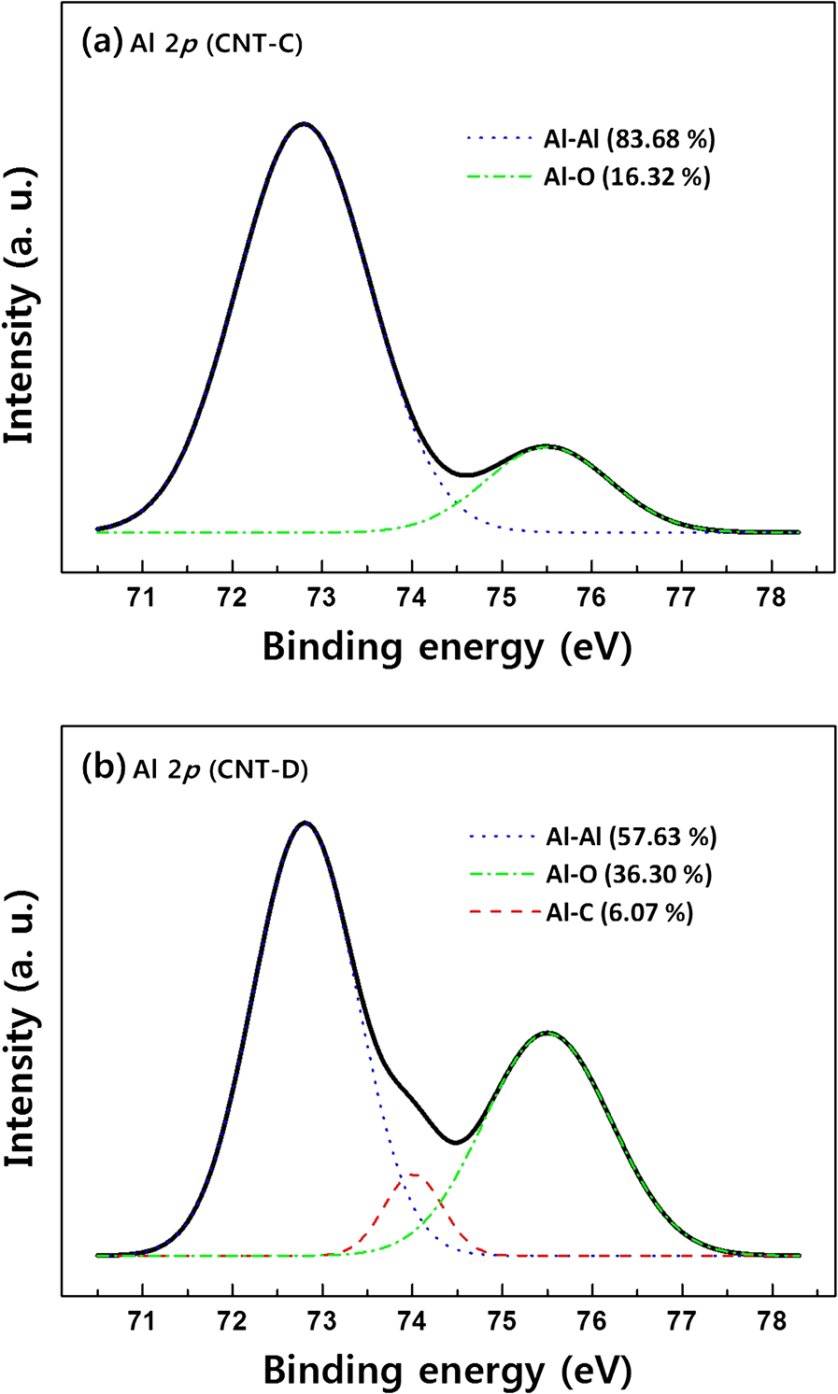
**The XPS spectra of the Al 2*p *states of the CNTs. (a)** The XPS result of the CNT-C emitter. **(b)** The XPS result of the CNT-D emitter.

**Figure 6:**
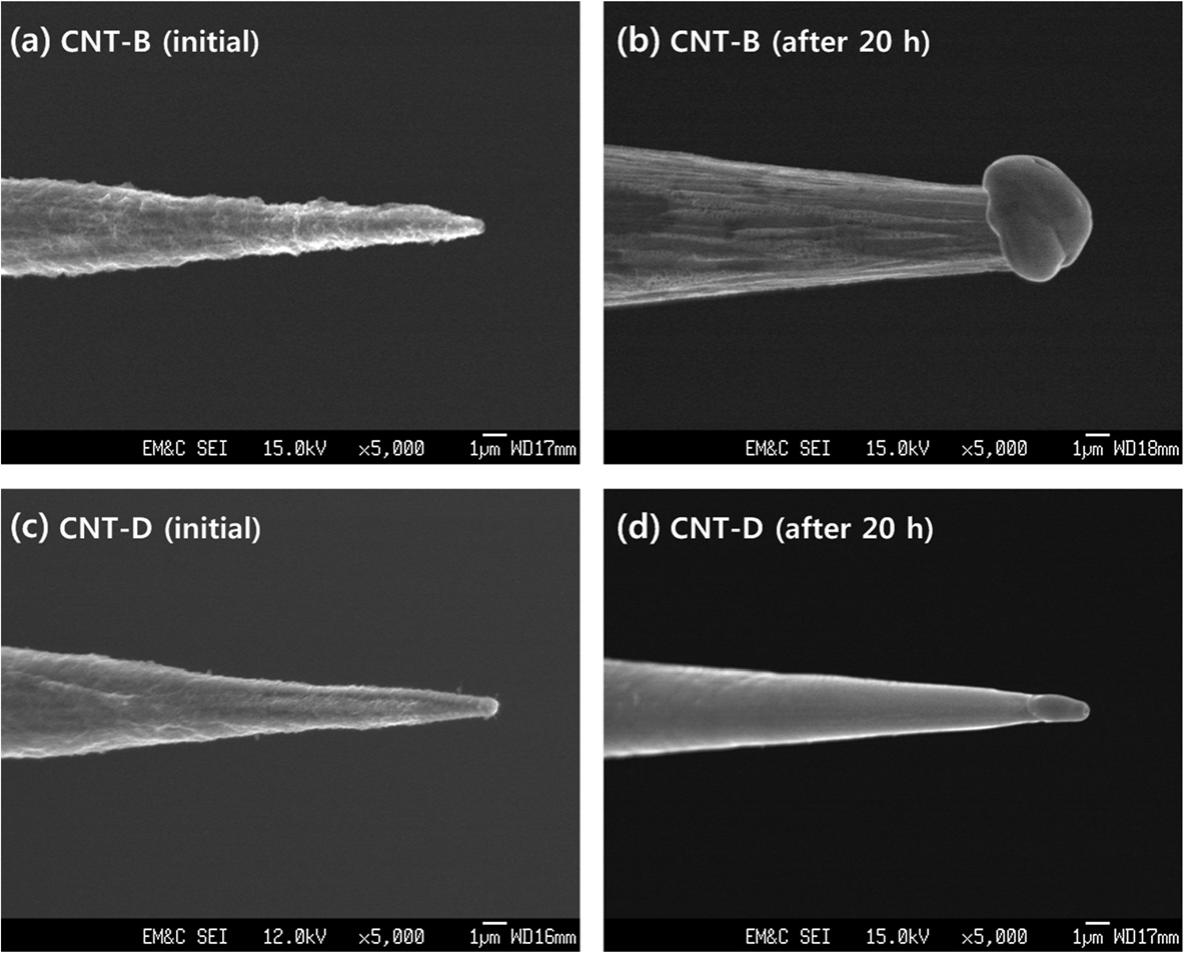
**The FESEM images before and after the stability test.** The morphologies of the CNT-B emitter, which were measured at the **(a)** initial (i.e., before the stability test) and **(b)** final (i.e., after 20-h emission) stages of electron emission. The CNT-D emitter's morphologies measured at the **(c)** initial and **(d)** final stages of electron emission.

The electron emission stability of CNTs may depend on how strongly the CNTs adhere to the underlying substrates during operation. Figure 5a,b shows the XPS spectra of the Al 2*p* states for the CNT-C and CNT-D samples, respectively. Both of the CNTs had the peaks of Al-O bonds at 75.5 eV as well as the relatively strong peaks of Al-Al metallic bonds at 72.8 eV. The peak intensity of the Al-O bonds was increased after thermal treatment, indicating that the oxidation of Al atoms was thermally activated [22]. The surface layers composed of the Al-O bonds may prevent the CNTs from being damaged by the ionized particles [12] during electron emission and also suppress the Joule heat [23] which may occur mainly near the summit part of the conical-shaped emitter. This was confirmed by the FESEM images of the CNT samples, which were measured at both their initial and final stages of electron emission, which are displayed in Figure 6. The CNT-B revealed that its summit part melted due to the prolonged electron emission, and the conical shape of the emitter summit disappeared, as shown in Figure 6b. In contrast, the CNT-D emitter maintained its morphology of having a conical shape even after 20 h of operation, as shown in Figure 6d. In the Al 2*p* XPS spectra of the CNT-D, furthermore, an additional peak at 74.0 eV due to the Al-C bonds was observed, as shown in Figure 5b. This may imply that the Al atoms incorporated in the Al interlayers were covalently bonded with the C atoms incorporated in the CNTs. This also indicates that coating of Al interlayer may provide the CNTs the additional chemical forces due to the Al-C interactions when the CNTs were thermally treated. It can be concluded, therefore, that the chemical bonds such as Al-O and Al-C may strengthen the adhesion between the CNTs and the substrates, eventually leading to the enhanced long-term emission stabilities of the CNTs.

## Conclusions

The conical-type CNT-based field emitters were fabricated using the EPD method. Substantially, enhanced emission characteristics, such as lower turn-on voltage and higher emission currents, were obtained by thermally treating the CNTs. From the FESEM observations as well as from the electrical measurements of emission characteristics, the thermal treatment barely affected the CNTs' surface morphologies and field enhancement factors. The observations of the Raman spectra confirmed that the improved emission characteristics of the thermally treated CNTs were ascribed to their higher degrees of crystallinities. In addition, the long-term emission stabilities of the CNTs were significantly ameliorated by coating Al interlayers prior to the deposition of CNTs. The CNTs, when deposited on the Al interlayers and thermally treated, exhibited highly stable electron emission behaviors without any significant degradation of emission currents even after 20 h of operation. The XPS results indicated that the improved adhesion of CNT-D was ascribed to the increase of Al-O bonds and the creation of Al-C bonds by thermal treatment. This may diminish the possibility of electric arcing at the W tip and also enhance the W tip's robustness against melting, which may eventually lead to the improved long-term emission stability of the CNTs. It was also reported by our previous work [14] that the emission stabilities of CNTs deposited on the W tips coated with Hf interlayer were improved only when the CNTs were thermally treated. This was due to the formation of carbide bonds (Hf-C) at elevated temperature. In this study, the CNTs using Al interlayers showed that the enhanced emission stabilities were observed not only for the thermally annealed CNTs but also for the as-deposited CNTs without thermal treatment. This was because oxide bonds (Al-O) already existed in the as-deposited CNTs, while carbide bonds (Al-C) were observed for the thermally annealed CNTs.

## Competing interests

The authors declare that they have no competing interests.

## Authors' contributions

BJK, JPK, and JSP have made substantial contributions to the conception, acquisition, and interpretation of data. All authors have been involved in drafting the manuscript and approved the final manuscript.

## Authors' information

BJK is currently a Ph.D. student of Electronic Systems Engineering Department in Hanyang University. His research focuses on the application of carbon nanotube in X-ray system and transparent conductive films. JPK, Ph.D., is currently working in Health & Medical Equipment Business Team, Samsung Electronics. JSP, Ph.D., is a professor of Electronic Systems Engineering Department in Hanyang University. He is currently interested in flexible transparent displays and carbon and related nano-devices.
